# Court diversion for those with psychosis and its impact on re-offending rates: results from a longitudinal data-linkage study

**DOI:** 10.1192/bjo.2018.71

**Published:** 2019-01-10

**Authors:** Olayan Albalawi, Nabila Zohora Chowdhury, Handan Wand, Stephen Allnutt, David Greenberg, Armita Adily, Azar Kariminia, Peter Schofield, Grant Sara, Sarah Hanson, Colman O'Driscoll, Tony Butler

**Affiliations:** Kirby Institute, University of New South Wales, Australia; Tabuk University, Department of Statistics, Science Faculty, Saudi Arabia; Biostatistician, Kirby Institute, University of New South Wales, Australia; Private Psychiatrist and Conjoint Lecturer, University of New South Wales, Australia; Director, New South Wales State-wide Clinical Court Liaison Service, New South Wales Justice and Forensic Mental Health Network; and Conjoint Lecturer, University of New South Wales, Australia; Research Associate, Kirby Institute, University of New South Wales, Australia; Senior Lecturer, Kirby Institute, University of New South Wales, Australia; Director, Neuropsychiatry Services, Hunter New England Mental Health, Australia; Director, InforMH, NSW Ministry of Health; and Clinical Associate Professor, University of Sydney Northern Clinical School, Australia; Director, Quality and Safeguards, Social Policy Group, New South Wales Department of Premier and Cabinet; and Juris Doctor, Mental Health Commission of New South Wales, Australia; Executive Director, Lifeline Australia; and Conjoint Lecturer, University of New South Wales, Australia; Program Head, Justice Health Research Program, Kirby Institute, University of New South Wales, Australia

**Keywords:** Court diversion, psychosis, offending

## Abstract

**Background:**

With significant numbers of individuals in the criminal justice system having mental health problems, court-based diversion programmes and liaison services have been established to address this problem.

**Aims:**

To examine the effectiveness of the New South Wales (Australia) court diversion programme in reducing re-offending among those diagnosed with psychosis by comparing the treatment order group with a comparison group who received a punitive sanction.

**Method:**

Those with psychoses were identified from New South Wales Ministry of Health records between 2001 and 2012 and linked to offending records. Cox regression models were used to identify factors associated with re-offending.

**Results:**

A total of 7743 individuals were identified as diagnosed with a psychotic disorder prior to their court finalisation date for their first principal offence. Overall, 26% of the cohort received a treatment order and 74% received a punitive sanction. The re-offending rate in the treatment order group was 12% lower than the punitive sanction group. ‘Acts intended to cause injury’ was the most common type of the first principal offence for the treatment order group compared with the punitive sanction group (48% *v.* 27%). Drug-related offences were more likely to be punished with a punitive sanction than a treatment order (12% *v.* 2%).

**Conclusions:**

Among those with a serious mental illness (i.e. psychosis), receiving a treatment order by the court rather than a punitive sanction was associated with reduced risk for subsequent offending. We further examined actual mental health treatment received and found that receiving no treatment following the first offence was associated with an increased risk of re-offending and, so, highlighting the importance of treatment for those with serious mental illness in the criminal justice system.

As significant numbers of individuals in the criminal justice system are known to have mental illnesses, mental health courts, problem-solving courts, court-based diversion programmes and liaison services have been established in response to this.^1^^,^^2^ Evaluations of such programmes, many conducted in the USA, have found they have positive outcomes in terms of reductions in re-offending, improvement in social outcomes and economic benefits.^3^^,^^4^ In Australia court diversion/liaison schemes have expanded over the past 18 years.^5^^–^^8^ The New South Wales' (NSW) State-wide Community and Court Liaison Service (SCCLS) was established in 1999 under the Mental Health [Forensic Provisions] Act to assist magistrates to identify mentally ill offenders charged with less serious criminal offences and provide the option of diverting defendants away from the criminal justice system to in-patient or community psychiatric care.^9^

The objective of this study was to examine the effectiveness of diversion of individuals with psychosis in NSW between 2001 and 2012 by comparing those receiving treatment orders with a comparison group receiving punitive sanctions. We examined factors associated with the incidence of re-offending by psychosis type, offence, sociodemographic factors and mental health service contact.

## Method

### Data sources

Diagnostic information was retrieved from the NSW Ministry of Health's Admitted Patient Data Collection (APDC) and the Emergency Department Data Collection (EDDC). The APDC covered the period 1 July 2001 to 31 December 2012, and the EDDC 1 January 2005 to 31 December 2012. Both the APDC and EDDC contain details of both primary and secondary diagnoses of health records, which were coded using ICD-9 and ICD-10,^10^^,^^11^ episode start date, episode end date, hospital type, country of birth, Aboriginal, marital status, gender, date of birth. Data on deaths for the cohort were extracted from the NSW Registry of Births, Deaths and Marriages (RBDM) for the period 1 July 2001 to 31 December 2012 covering date of death and age at death in years, and Mental Health Ambulatory Data Collection covered community mental health treatment in the period following the court finalisation date for the first offence.

Individuals with psychosis were either diverted from the courts into treatment (treatment order group) and management under Section 32 and Section 33 of the Mental Health (Forensic Provisions) Act between 2001 and 2012 or received a punitive sanction (i.e. bond, community order, fine, probation and suspended sentence) and were identified from the NSW Bureau of Crime Statistics and Research's Re-offending Database (RoD). Other data extracted from the RoD included: gender, Aboriginality status, date of birth, offence date, date of the finalisation of contact with the court, date of death, offence type and principal penalty. Minor traffic infringements were excluded from the offence data.

Mental health treatment contacts were identified from the APDC, EDDC and Mental Health Ambulatory Data Collection after the court finalisation date for the first offence and before the second offence date or the end of the study period on 31 December 2015 for those who did not commit a second offence.

### Record linkage process

Record linkage between the health (APDC and EDDC), RoD and RBDM data sources was conducted by the NSW Ministry of Health's Centre for Health Record Linkage (CHeReL) using probabilistic record linkage methods and *ChoiceMaker* software. Identifying information such as name, address, date of birth and gender for each data-set is included in the Master Linkage Key. No health data are used in the linkage process. Once the linkages were finalised, the CHeReL created a project person number for each person identified in the linkage, and assigned this project person number to the RoD, APDC, EDDC, RBDM death records. These data sources were merged by using the project person number.

### Cohort

Those with a pre-existing diagnosis of psychosis prior to their court finalisation date for the first offence were identified and formed the basis for the cohort examined in the analysis. ICD-9 and ICD-10 primary and secondary diagnosis codes for psychosis were obtained from the APDC and EDDC.

The following ICD-9 and ICD-10 codes were used to define psychosis: schizophrenia and related psychoses (F20.1–F20.6, F20.8, F20.9, F22.0, F22.8, F22.9, F23.2, F23.3, F23.9, F25.0-F25.2, F25.9, F29 and 295); affective psychosis (F30.2, F31.2, F31.5, F32.3 F33.3, 296.8 and 296.9), and substance-related psychosis (F10.5, F13.5, F14.5, F15.5, F16.5, F19.5, 291 and 292).^10^^,^^11^

Socio-Economic Indexes for Areas (SEIFA) is a measure derived from the statistical local area, which is a geographic area based on local government area. One SEIFA measure is the Index of Relative Socio-economic Advantage and Disadvantage, which is based on national census data using income, education, employment, occupation and housing. Areas are ranked with the lowest indicating the most disadvantage and highest the most advantage for an area. For the purpose of the current analysis, we used the SEIFA score based on the area of residence at the time of the most recent diagnosis, and categorised the ten categories into two representing ‘disadvantaged’ (SEIFA score 1–5) and ‘advantaged’ (SEIFA score 6–10).^12^

### Offending behaviour categories

Criminal charges were classified as either violent or non-violent based on the Australian and New Zealand Standard Offence Classification system.^13^

### Outcome measurements

The outcomes of interest were: (a) time to the second offence following the first principal offence in the data-set by sanction type (i.e. treatment order versus punitive sanction) for men and women, offence type (violent and non-violent) and diagnostic group; and (b) the predictors of second offence.

### Statistical analysis

Characteristics of the study population at the time of diagnosis were summarised using descriptive statistics frequencies and proportions for the categorical variables and median and interquartile range (IQR) for the continuous variables. χ^2^ tests were used to examine the difference between the treatment order and punitive sanction groups among men and women, and Aboriginal and non-Aboriginal groups.

The main outcome variable was second offence following the first principal offence. For this (survival) analysis, time to second offence was calculated as time (years) from the court finalisation date for the first principal offence record in the data to the second offence. Univariable and multivariable Cox regression models with random effect were used to identify significant predictors of the second offence after accounting for potential heterogeneity because of socioeconomic status (SEIFA).^14^^,^^15^ To account for periods of time in hospital when an individual cannot be at risk of re-offending we subtracted this time from the total follow-up time. Those who were sentenced to prison as the outcome of the first offence were excluded from the analysis.

Treatment received by those in both the treatment order (diverted) and comparison groups between the finalisation date for the first offence and second offence date (or end of the study for those who did not re-offend) was determined by examining all contacts with community mental health services (Mental Health Ambulatory Data Collection), emergency department contacts associated with psychosis (EDDC) and hospital admissions associated with psychosis (APDC). As community mental health contacts do not always involve a clinical interaction *per se*, we identified those codes in the Mental Health Ambulatory Data Collection system likely to involve a clinical component rather than those representing administrative tasks.

The current study considered the following factors as potential predictors for second offence: court outcome (Section 32/Section 33 treatment order/type of punitive sanction), psychosis type (schizophrenia and related psychoses, substance-related psychosis versus affective psychosis), age at the first principal offence (<18 years, 18–26 years, 26–35 years, 36–45 years versus ≥46 years), Aboriginal status (no versus yes, unknown), marital status (married versus other), country of birth (Australia versus other, unknown), contact with mental health services between the court finalisation date for the first principal offence and second offence (no versus yes) and type of first principal offence (violent versus non-violent). Crude re-offending incidence rates were presented using Kaplan–Meier curves for the treatment order and punitive sanction groups by gender, psychosis type and offence.

### Ethics

Approvals for the project were independently received from the following: NSW Population & Health Services Research Ethics Committee (HREC/15/CIPHS/17); Justice Health and Forensic Mental Health Network (G324/14); Corrective Services NSW (D15/138715); and the NSW Aboriginal Health and Medical Research Council (1089/15).

## Results

### Characteristics of the treatment order and comparison (punitive) groups

A total of 7743 individuals were identified who had been diagnosed with a psychotic disorder before the court finalisation date for their first principal offence. Of these 1996 (26%) received a treatment order and diverted from criminal justice system under Section 32 or Section 33 (prior to 2004, court outcomes were simply coded as ‘dismissed by the lower court due to mental illness’), and 5747 (74%) individuals received a punitive sanction (47% bond, 44% fine, 2% community order, 6% suspended sentence, and <1% probation) for the first principal offence. Of the 1996 individuals who received a treatment order, and for whom we had information on the type of order (*n* = 1469), 85% received a Section 32 order and 15% received a Section 33 order.

Between 2001 and 2012, 1996 (26%) individuals with psychosis appearing in the NSW courts where the SCCLS was in operation, received a treatment order. The SCCLS currently operates in 22 courts across NSW growing from 7 courts in 2001.^16^ There was no significant difference in the proportion of men and women with psychosis who received a treatment order over time (26% of those with psychosis among men and 25% among women, *P* = 0.471). However, a significantly lower proportion of Aboriginal people received a treatment order compared with non-Aboriginal people (7% *v.* 11%, *P* < 0.001) (Table 1).

**Table 1:** Characteristics of diversion programme group versus punitive sanction group

	Total (*n* = 7743)			Men (*n* = 5643, 73%)			Women (*n* = 2100, 27%)		
	Treatment order (*n *= 1996, 26%)	Punitive sanction (*n* = 5747, 74%)	*P*	Treatment order (*n* = 1467, 26%)	Punitive sanction (*n* = 4176, 74%)	*P*	Treatment order (*n* = 529, 25%)	Punitive sanction (*n* = 1571, 75%)	*P*
Age at the first offence									
<18 years	19 (1)	50 (1)	<0.001	12 (<1)	20 (1)	0.004	7 (1)	30 (2)	0.002
18–25 years	393 (20)	1193 (21)		316 (22)	870 (21)		77 (15)	323 (21)	
26–35 years	656 (33)	1979 (34)		481 (33)	1453 (35)		175 (33)	526 (33)	
36–45 years	513 (26)	1582 (28)		361 (25)	1142 (27)		152 (29)	440 (28)	
≥46 years	415 (21)	943 (16)		297 (20)	691 (17)		118 (22)	252 (16)	
Aboriginal									
Yes	148 (7)	625 (11)	<0.001	94 (6)	380 (9)	0.004	54 (10)	245 (16)	0.001
No	1662 (83)	4514 (79)		1244 (85)	3406 (82)		418 (79)	1108 (71)	
Unknown	186 (9)	608 (11)		129 (9)	390 (9)		57 (11)	218 (14)	
Marital status									
Married	241 (12)	718 (12)	0.625	149 (10)	466 (11)	0.289	92 (17)	252 (16)	0.468
Other	1755 (88)	5029 (88)		1318 (90)	3710 (89)		437 (83)	1319 (84)	
Country of birth									
Australia	1355 (68)	3988 (69)	<0.001	986 (67)	2864 (69)	0.007	369 (70)	1124 (71.5)	<0.001
Other	429 (21)	910 (16)		324 (22)	695 (17)		105 (20)	215 (14)	
Missing	212 (11)	849 (15)		157 (11)	617 (15)		55 (10)	232 (15)	
Psychosis type									
Schizophrenia and related psychoses	1608 (81)	3743 (65)	<0.001	1191 (81)	2773 (66)	<0.001	417 (79)	970 (62)	<0.001
Affective psychosis	241 (12)	555 (10)		169 (12)	362 (9)		72 (14)	193 (12)	
Substance-related psychosis	147 (7)	1449 (25)		107 (7)	1041 (25)		40 (8)	408 (26)	
Socio-Economic Indexes for Areas									
Advantaged (6–10)	1208 (61)	3065 (53)	<0.001	870 (59)	2220 (53)	<0.001	338 (64)	845 (54)	<0.001
Disadvantaged (1–5)	788 (39)	2682 (47)		597 (41)	1956 (47)		191 (36)	726 (46)	

The treatment order and punitive sanction groups were similar with regard to their age at the time of first offence: 34 years (IQR 27–44) and 34 years (IQR 27–42), respectively. However, schizophrenia and related psychoses prior to the court finalisation date was more common in the treatment order than the punitive sanction group (81% *v.* 65%) and substance-related psychosis more common in the comparison (punitive) group (25% *v.* 7%) (Table 1). There was a shorter time-lag between the most recent diagnosis and the court finalisation date for the first principal offence in the diversion group compared with the punitive sanction group – median number of days: 114 days (IQR 49–248) versus 258 days (IQR 86–738).

### Offence type by treatment order and comparison (punitive) group

Overall, the most common first principal offence was ‘Acts intended to cause injury’, which was significantly higher in the treatment order group compared with the punitive sanction group (48% *v.* 27%) (Table 2). Further when we grouped violent offences, a higher proportion of this group received a treatment order rather than a punitive sanction (56% *v.* 38%) (Table 2). Those committing drug-related offences were more likely to receive a punitive sanction than a treatment order (12% *v.* 2%). Few homicides and sexual offences occurred in the cohort, which is consistent with more serious matters being heard in higher courts.

**Table 2:** Principal offence type (first offence) by outcome (treatment order versus punitive sanction) and gender

Offence Type (ANZSOC code)	Treatment order group			*P*	Punitive sanction group			*P*
	Total (*n *= 1996, 100%)	Men (*n *= 1467, 73%)	Women (*n *= 529, 27%)		Total (*n *= 5737, 100%)	Men (*n *= 4173, 73%)	Women (*n *= 1564, 27%)	
Homicide (0111–0132)	0	0	0	<0.001	9 (<1)	4 (<1)	5 (<1)	<0.001
Acts intended to cause injury (0211–0299)	952 (48)	702 (48)	250 (47)		1538 (27)	1097 (26)	441 (28)	
Sexual offences (0311–0329)	36 (2)	35 (2)	1 (<1)		41 (1)	39 (<1)	2 (<1)	
Dangerous or negligent acts (0411–0499)	71 (4)	48 (3)	23 (4)		467 (8)	338 (8)	129 (8)	
Abduction, harassment (0511–0532)	48 (2)	41 (3)	7 (1)		61 (1)	46 (1)	15 (1)	
Robbery, extortion (0611–0621)	16 (1)	12 (1)	4 (1)		42 (1)	36 (1)	6 (<1)	
Burglary (0711)	69 (3)	57 (4)	12 (2)		91 (2)	76 (2)	15 (1)	
Theft (0811–0841)	169 (8)	109 (7)	60 (11)		687 (12)	421 (10)	266 (17)	
Fraud, deception (0911–0999)	49 (2)	27 (2)	22 (4)		203 (4)	124 (3)	79 (5)	
Drugs offences (1011–1099)	33 (2)	25 (2)	8 (2)		668 (12)	541 (13)	127 (8)	
Weapons and explosives offences (1111–1129)	14 (1)	12 (1)	2 (<1)		73 (1)	64 (2)	9 (1)	
Property/environmental damage (1211–1229)	188 (9)	131 (9)	57 (11)		440 (8)	330 (8)	110 (7)	
Public order offences (1311–1334)	135 (7)	117 (8)	18 (3)		508 (9)	397 (10)	111 (7)	
Traffic offences (1411–1441)	11 (1)	9 (1)	2 (<1)		214 (4)	157 (4)	57 (4)	
Offences against justice/govt. procedures (1511–1569)	198 (10)	140 (10)	58 (11)		610 (11)	446 (11)	164 (10)	
Miscellaneous offences (1611–1699)	7 (<1)	2 (<1)	5 (1)		85 (1)	57 (1)	28 (2)	
Type of first offence				0.197				0.457
Non-violent offence	873 (44)	629 (43)	244 (46)		3579 (62)	2613 (63)	966 (62)	
Violent offence	1123 (56)	838 (57)	285 (54)		2158 (38)	1560 (38)	598 (38)	

ANZSOC, Australian and New Zealand Standard Offence Classification.

Among those who had committed a non-violent first principal offence, a lower proportion received a treatment order than a punitive sanction (44% *v.* 62%). Similarly, in both men and women those who committed non-violent offences were less likely to receive a treatment order (men 43% *v.* 63%; women 46% *v.* 62%) (Table 2).

The most common non-violent offence types in the diversion group were offences against justice/government procedures (23%), property and environmental damage (22%) and public order (15%), whereas among the punitive sanction group the most common non-violent offence types were drugs (19%), theft (19%) and offences against government procedures (17%). The most common type of violent offence committed for the first principal offence in both the treatment order and punitive sanction groups were acts intended to cause injury (85% and 71%, respectively). Theft was more frequent in women compared with men in the treatment order group (11% *v.* 7%) (Table 2).

### Incidence of re-offending

**Fig. 1:**
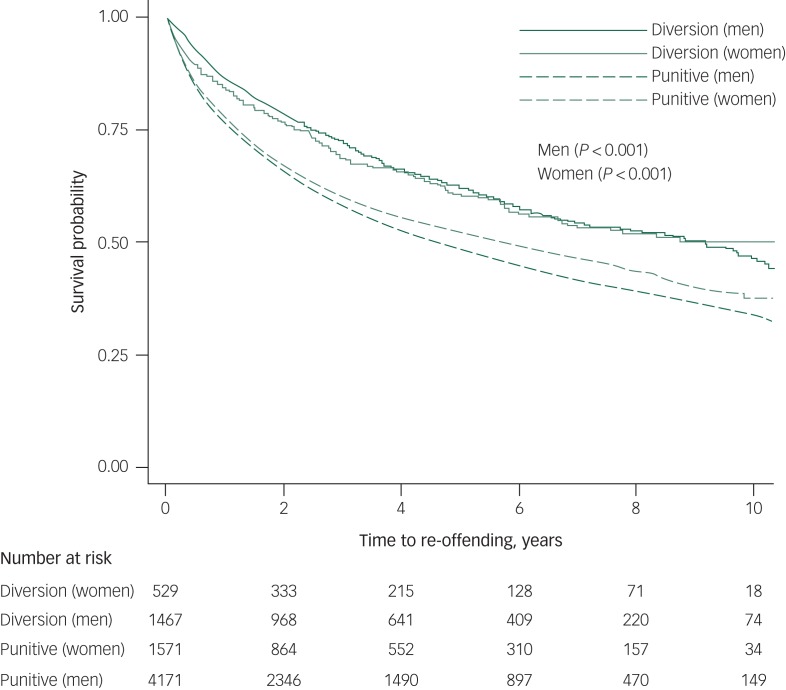
Kaplan–Meier curves for diversion program versus punitive sanction groups by gender.

Re-offending in the treatment order group was 12% lower than the punitive sanction group (38% *v.* 50%). The median number of years between the finalisation date for the first principal offence and the second offence or the end study date for the diversion group was 5.1 years (IQR 2.2–8.4) and 4.0 year (IQR 1.1–7.6) for the punitive sanction group. These findings are further supported by survival analysis that indicated that there was a significant difference in the time to re-offence from the first recorded offence in the data-set between those men and women who received a treatment order and those who receive a punitive sanction (*P* < 0.001 log-Rank test) (Fig. 1). Re-offending rates ranged from 6.8 per 100 person-years (95% CI 6.3–7.4) (for men in the treatment order group) to 11.1 per 100 person-years (95% CI 10.6–11.6) for men in the punitive sanction group and from 6.9 per 100 person-years (95% CI 6.0–8.0) for women in the treatment order group to 9.6 (95% CI 8.9–10.4) for women in the punitive sanction.

Crude incidence rates for re-offending were also calculated by diagnostic groups. Re-offending rates ranged from 5.3 per 100 person-years (95% CI 4.2–6.6) for those with affective psychosis in the treatment order group to 7.8 per 100 person-years (95% CI 6.9–8.9) for those with affective psychosis in the punitive sanction group. For individuals with schizophrenia and related psychoses re-offending rates ranged from 7.0 per 100 person-years (95% CI 6.5–7.6) in the treatment order group to 10.6 per 100 person-years (95% CI 10.1–11.1) in the punitive sanction group. Moreover, re-offending rates for individuals with substance-related psychosis ranged from 8.1 per 100 person-years (95% CI 6.4–10.4) in the treatment order group to 12.3 per 100 person-years (95% CI 11.4–13.2) in the punitive sanction group.

**Fig. 2:**
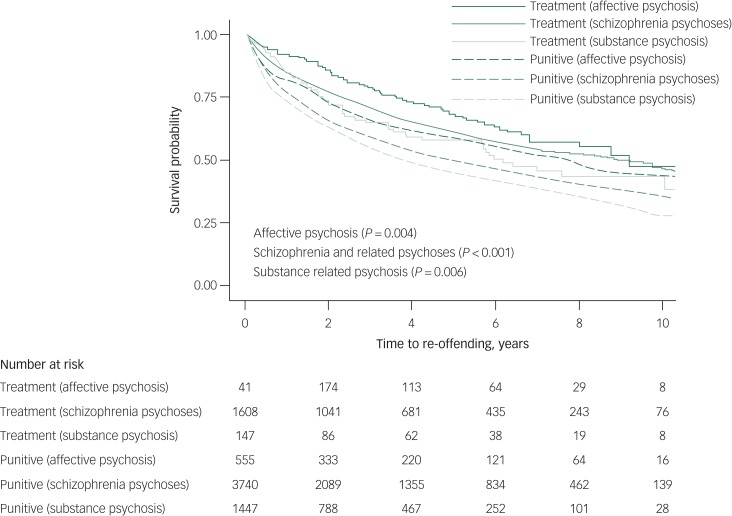
Kaplan–Meier curves for diversion program versus punitive sanction groups by psychosis type.

**Fig. 3:**
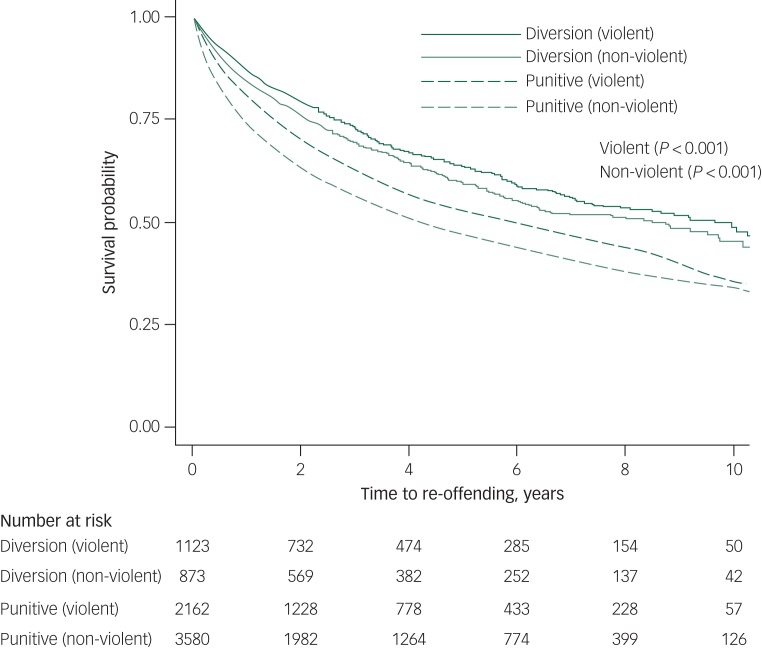
Kaplan–Meier curves for diversion program versus punitive sanction groups by first principal offence type.

Treatment orders were least effective in terms of reducing re-offending in those with a diagnosis of substance-related psychosis (*P* = 0.006), and most effective for those diagnosed with affective psychosis (*P* < 0.001) (Fig. 2). Those with substance-related psychosis and schizophrenia and related psychoses had the lowest survival probability with regards to re-offending. Re-offending rates ranged from 7.4 per 100 person-years (95% CI 6.7–8.2) for individuals with a non-violent first offence in the treatment order group to 11.6 per 100 person-years (95% CI 11.1–12.1) for those with a non-violent first offence in the punitive sanction group. The re-offending rates ranged from 6.4 per 100 person-years (95% CI 5.8–7.1) for individuals with a violent first offence in the treatment order group to 9.3 per 100 person-years (95% CI 8.7–9.9) for those with a violent first offence in the punitive sanction group. Receiving a treatment order for both violent or non-violent offences was significantly associated with a decreased risk of re-offending compared with the punitive sanction group (Fig. 3).

After adjusting the results for age, marital status, country of birth and psychosis types, the treatment order group were less likely to commit a second offence compared with the punitive sanction group (adjusted hazard ratio (HR) = 0.68, 95% CI 0.62–0.74, *P* < 0.001) (Table 3). Individuals diagnosed with substance-related psychosis had a higher risk of re-offending compared with those diagnosed with affective psychosis regardless of whether they were in the treatment order or punitive sanction group (adjusted HR = 1.46, 95% CI 1.27–1.67) (Table 3). In addition, those who were diagnosed with schizophrenia and related psychoses had a higher risk of re-offending compared with those with affective psychosis (adjusted HR = 1.19, 95% CI 1.05–1.34).

**Table 3:** Adjusted hazard ratios (HRs)^a^ for re-offending among men and women

	Overall (*n* = 7743)			Men (*n* = 5643; 73)			Women (*n* = 2100; 27)		
	*n* (%)	Adjusted HR (95% CI)	*P*	*n* (%)	Adjusted HR (95% CI)	*P*	*n* (%)	Adjusted HR (95% CI)	*P*
Group									
Punitive sanction	5747 (74)	1		4176 (74)	1		1571 (75)	1	
Treatment order	1996 (26)	0.68 (0.62–0.74)	<0.001	1467 (26)	0.65 (0.59–0.72)	<0.001	529 (25)	0.78 (0.66–0.92)	<0.001
Psychosis type									
Affective psychosis	796 (10)	1		531 (9)	1		265 (13)	1	
Schizophrenia and related psychoses	5351 (69)	1.19 (1.05–1.34)	0.005	3964 (70)	1.15 (1.00–1.33)	0.058	1387 (66)	1.32 (1.06–1.64)	0.014
Substance related psychosis	1596 (21)	1.46 (1.27–1.67)	<0.001	1148 (20)	1.47 (1.25–1.73)	<0.001	448 (21)	1.41 (1.09–1.82)	0.008
Age at the first offence									
<18 years	69 (1)	2.26 (1.65–3.09)	<0.001	32 (1)	1.93 (1.20–3.10)	0.007	37 (2)	2.73 (1.77–4.23)	<0.001
18–25 years	1586 (20)	1.57 (1.40–1.77)	<0.001	1186 (21)	1.53 (1.34–1.76)	<0.001	400 (19)	1.73 (1.37–2.19)	<0.001
26–35 years	2635 (34)	1.64 (1.47–1.83)	<0.001	1934 (34)	1.62 (1.43–1.84)	<0.001	701 (33)	1.76 (1.42–2.19)	<0.001
36–45 years	2095 (27)	1.34 (1.19–1.50)	<0.001	1503 (27)	1.37 (1.19–1.56)	<0.001	592 (28)	1.28 (1.02–1.61)	0.034
46+ years	1358 (18)	1		988 (18)	1		370 (18)	1	
Aboriginal									
No	6176 (80)	1		4650 (82)	1		1526 (73)	1	
Yes	773 (10)	1.85 (1.68–2.03)	<0.001	474 (8)	1.73 (1.54–1.94)	<0.001	299 (14)	2.13 (1.82–2.50)	<0.001
Unknown	794 (10)	0.08 (0.06–0.11)	<0.001	519 (9)	0.08 (0.06–0.12)	<0.001	275 (13)	0.08 (0.05–0.13)	<0.001
Marital status									
Married	959 (12)	1		615 (11)	1		344 (16)	1	
Other	6784 (88)	1.21 (1.09–1.35)	<0.001	5028 (89)	1.09 (0.96–1.24)	0.195	1756 (84)	1.51 (1.23–1.84)	<0.001
Country of birth									
other	1339 (17)	1		1019 (18)	1		320 (18)	1	
Australia	5343 (69)	1.18 (1.08–1.30)	<0.001	3850 (68)	1.21 (1.09–1.34)	<0.001	1493 (71)	1.15 (0.95–1.40)	0.158
Unknown	1061 (14)	1.27 (1.12–1.45)	<0.001	774 (14)	1.24 (1.08–1.45)	0.004	287 (14)	1.37 (1.05–1.79)	0.018
First principal offence type									
Violent	3288 (43)	1		2399 (43)	1		889 (42)	1	
Non-violent	4455 (57)	1.20 (1.12–1.28)	<0.001	3244 (57)	1.24 (1.15–1.34)	<0.001	1211 (58)	1.07 (0.94–1.23)	0.279

a. Adjusted by age, marital status, country of birth and psychosis type.

Those offenders who were less than 18 years old at the time of the first offence had a higher risk of committing a second offence compared with those over 46 years (adjusted HR = 2.26, 95% CI 1.65–3.09, *P* < 0.001). In addition, the results showed that those of Aboriginal heritage had an 85% higher risk of re-offending compared with the non-Aboriginal group (adjusted HR = 1.85, 95% CI 1.68–2.03, *P* < 0.001) (Table 3).

Table 4 shows a similar pattern in terms of factors associated an increased hazard of re-offending between violent and non-violent offenders for their first principal offence.

**Table 4:** Adjusted hazard ratios (HRs)^a^ for re-offending among non-violent and violent for the first principal offence type (violent and non-violent)

	Non-violent (*n* = 4455)			Violent (*n* = 3288)		
	*n* (%)	Adjusted HR (95% CI)	*P*	*n* (%)	Adjusted HR (95% CI)	*P*
Group						
Punitive sanction	3582 (80)	1		1123 (34)	1	
Treatment order	873 (20)	0.69 (0.62–0.78)	<0.001	2165 (66)	0.74 (0.66–0.84)	<0.001
Psychosis type						
Affective psychosis	413 (9)	1		383 (12)	1	
Schizophrenia and related psychoses	3020 (68)	1.14 (0.98–1.34)	0.099	2331 (71)	1.25 (1.04–1.51)	0.016
Substance related psychosis	1022 (23)	1.33 (1.12–1.59)	0.001	574 (17)	1.50 (1.21–1.86)	<0.001
Age at the first offence						
<18 years	25 (1)	2.30 (1.40–3.76)	0.001	44 (1)	2.24 (1.47–3.39)	<0.001
18–25 years	944 (21)	1.63 (1.41–1.90)	<0.001	642 (20)	1.65 (1.36–2.00)	<0.001
26–35 years	1525 (34)	1.76 (1.53–2.03)	<0.001	1110 (34)	1.56 (1.30–1.86)	<0.001
36–45 years	1196 (27)	1.41 (1.22–1.64)	<0.001	899 (27)	1.27 (1.05–1.53)	0.012
46+ years	765 (17)	1		593 (18)	1	
Aboriginal						
No	3606 (81)	1		2570 (78)	1	
Yes	438 (10)	1.77 (1.53–2.05)	<0.001	335 (10)	1.70 (1.50–1.91)	<0.001
Unknown	411 (9)	0.10 (0.06–0.15)	<0.001	383 (12)	0.08 (0.05–0.12)	<0.001
Marital status						
Married	466 (10)	1		493 (15)	1	
Other	3989 (90)	1.21 (1.04–1.40)	0.012	2795 (85)	1.13 (0.96–1.33)	0.155
Country of birth						
Other	717 (16)	1		622 (19)	1	
Australia	3126 (70)	1.09 (0.97–1.22)	0.159	2217 (67)	1.19 (1.03–1.38)	0.019
Unknown	612 (14)	0.98 (0.83–1.16)	0.802	449 (14)	1.33 (1.09–1.64)	0.006
Mental health treatment^b^						
No	1827 (41)	1		1225 (37)	1	
Yes	2628 (59)	1.16 (1.08, 1.24)	<0.001	2063 (63)	1.22 (1.14–1.31)	<0.001

a. Adjusted by age, marital status, country of birth and psychosis types.

b. Mental health treatment – 6 months following finalisation date.

### Treatment and re-offending

Mental health service treatment frequency following the court finalisation date was examined by looking at all in-patient or out-patient contacts with mental health services occurring between the court finalisation date for the first offence and the second offence date or the end of the study (in those individuals who did not commit a second offence). The proportion of those with a record of contact with mental health services following the court finalisation date for the first offence was higher in the treatment order group versus the punitive sanction group (91% *v.* 80%) (Table 5).

**Table 5:** Adjusted hazard ratios (HRs)^a^ for re-offending among treatment order and punitive sanction groups and contact with mental health services

	Treatment order group (*n* = 1996)			Punitive sanction group (*n* = 5747)		
	*n* (%)	Adjusted HR (95% CI)	*P*	*n* (%)	Adjusted HR (95% CI)	*P*
Contact with mental health services						
Yes	1820 (91)	1		4606 (80)	1	
No	176 (9)	2.00 (1.61–2.50)	<0.001	1141 (20)	2.54 (2.34–2.76)	<0.001
Total contacts with mental health services						
0	176 (9)	4.94 (3.71–6.58)	<0.001	1141 (20)	6.92 (5.79–8.28)	<0.001
1–5	295 (15)	4.72 (3.65–6.09)	<0.001	1314 (23)	4.11 (3.44–4.20)	<0.001
6–20	286 (14)	3.74 (2.88–4.85)	<0.001	1028 (18)	3.16 (2.63–3.81)	<0.001
21–60	300 (15)	3.41 (2.63–4.42)	<0.001	828 (14)	2.68 (2.21–3.25)	<0.001
61–200	432 (22)	1.88 (1.44–2.44)	<0.001	782 (14)	1.99 (1.63–2.42)	<0.001
≥201	507 (25)	1		654 (11)	1	

a. Adjusted by age, gender and psychosis types.

Overall, regardless of group (i.e. treatment order or punitive) those who had no contact with mental health services had an increased risk of re-offending, and the more treatment received was associated with a reduced risk of re-offending. Those in either the treatment order or punitive sanction groups who received no discernible treatment had the highest risk of re-offending (Table 5).

## Discussion

### Main findings

This study represents the most comprehensive analysis of a court diversion programme for those with a mental illness in Australia, and one of the few studies to examine court diversion for those with serious mental illness (psychosis) who had contact with the NSW public service and mental health treatment services. Our findings indicate that diversion into treatment is associated with a significantly lower rate of subsequent re-offending in men and women, regardless of offence type (violent or non-violent). Those with psychosis who received a treatment order had a 12% lower re-offending rate than those in the comparison group who received a punitive sanction such as a fine, community order or good behaviour bond. Overall, the effectiveness of receiving a treatment order increased – re-offending was 32% lower hazard than in the punitive sanction group – when adjusted for age, marital status, country of birth and psychosis type.

### Sentencing patterns

Between 2009 and 2011 the number of treatment orders was 50% lower in the Aboriginal group compared with the non-Aboriginal group. This may indicate that diversion programmes need to be culturally sensitive to Aboriginal people and further research is needed to examine this issue.

The study also provides potential insights into magistrates' sentencing patterns for different offender groups. Overall, almost three-quarters of those with a prior diagnosis of psychosis were given punitive sanctions rather than a treatment order. This suggests caution on the part of magistrates to divert those before the courts into mental health treatment. Diversion into treatment under Section 32 or Section 33 of the Mental Health Act is discretionary. Magistrates are not obliged to deal with the person under Section 32 or Section 33, even if expert evidence is available to support the presence of a mental illness/condition or developmental disability.^17^ In NSW, only those charged with offences regarded as summary offences and not strictly indictable offences are given the opportunity for diversion to the community. When a person is diverted into the general psychiatric mental health system, they are not referred into a specialist forensic psychiatric service and essentially become general mental health patient.

Once the magistrate has determined that an individual has a mental condition/illness or developmental disability they are required to consider whether diversion is ‘more appropriate’, with regard to factors such as the seriousness of the offence, the degree to which the condition contributed to the offence, the treatment and management plan and the likelihood of the person adhering to the treatment and management plan. Magistrates must also balance legal issues such as the need for punishment and deterrence, with the public interest in diverting the particular individual away from the criminal justice system and into treatment and management. In addition, magistrates have the option of dealing with the matter by way of a conditional bond that could include the expectation to pursue mental health treatment and in some cases be indistinguishable from a treatment order.

This relatively low diversion rate (26%, Table 1) for those with psychosis likely reflects the complexity of this decision-making process. The low diversion rate could also relate to a limited confidence that magistrates have in the preparedness of treating clinicians to inform the court when breaches of treatment plans occur and adherence to the treatment plan by those receiving a treatment order. However, only 9% of those given a treatment order had no record of contact with mental health services.

In addition, it is likely that a number of those who received a treatment order were followed up by private mental health practitioners rather than a public hospital or community mental health service. Private mental health practitioner records and general practitioner records are not part of the current linkage as these are held by the Commonwealth government as part of the Medicare system. Thus, it cannot be assumed that all of the 9% who were diverted failed to comply and this requires further investigation. These findings suggest that magistrates' discretionary application of diversion by treatment order warrants further investigation, particularly in light of our finding that engagement in treatment has a positive impact in terms of reducing subsequent offending.

#### Sentencing in relation to the timing of the diagnosis

Those who had a diagnosis of psychosis closer in time to the court finalisation date were more likely to receive a treatment order than those with a more remote diagnosis; this may relate to the former group being more mentally unwell and this being apparent to the magistrate and lawyers. The median lag between diagnosis and court finalisation date was 114 days in the diversion group versus 258 days in the punitive sanction group. It is possible that closer proximity between the diagnosis of psychosis and the court appearance may impress on the magistrate an apparent association between mental illness and offending resulting in a treatment order rather than a punitive sanction. However, this could also reflect the effectiveness of having an on-site court liaison service available to the court to provide a more immediate opinion and increase confidence in a forensic mental health opinion given the pressure on magistrates to finalise matters and clear court backlogs. It is possible that a treatment order is a means of disposing of cases fairly quickly. Recently, the NSW government invested AU$39 million dollars to appoint new judges to clear court backlogs in NSW.^18^

#### Handling of drug- and violence-related offences

The data suggest that magistrates are less likely to divert those with drug-related offences; these individuals were more likely to receive a punitive sanction than a treatment order (12% *v.* 2%). This pattern is further reinforced by looking at the diagnosis whereby those with a prior diagnosis of substance-related psychosis were more likely to receive a punitive sanction and not a treatment order (25% *v.* 7%). Of the 701 offenders appearing before the courts with a drug-related offence, only 33 (5%) received a treatment order.

This contrasts with violence-related offences whereby magistrates were more likely to divert this group into treatment and management (56% violent *v.* 38% non-violent). It is possible that magistrates divert individuals with psychosis with violent-related offences because they regard the violent behaviour as being driven by the psychosis and thus mitigating the level of responsibility. In contrast, in the drug-induced psychosis group, the violence is likely attributed to the drugs, not mental illness. It could be that magistrates perceive a nexus between illness and violence and have greater confidence in the effectiveness of treatment and management in diminishing the risk to the community in those with a non-drug-related psychosis as opposed to those with a drug-related psychosis. A further justification for this pattern may be that treatment orders appeared to be the least effective approach in terms of reducing re-offending in those with a diagnosis of psychosis because of psychoactive substance use.

There is a strong empirical association between re-offending and substance use in populations of offenders with psychosis in general. However, other factors that could be associated with the higher re-offending rate among those with substance-related psychosis in this study could include: a punitive sanction reducing the opportunity to become engaged in treatment and management; those found ‘guilty’ might experience discrimination from treatment providers and be less likely to follow treatment and management; differences in treatment and management modalities between mainstream psychiatric management and addiction-base management could also be relevant (for example, opioid substitution therapy that requires daily medication may be more demanding and so prone to non-adherence than antipsychotic medication that can be administered intramuscularly and last up to 4 weeks); there may be social bias in the courts and community.

### Limitations

Limitations of the current study include the extent to which the application of Section 32 or Section 33 orders might influence discretion by police in relation to charging individuals for subsequent incidents, but it is possible that greater latitude might be offered such individuals for their behaviour (relative to those who were punished rather than received treatment orders) to account for some of the differences in subsequent rates of court-determined re-offending. Furthermore, because of the observational nature of the study, we cannot exclude the possibility that punitive sanctions in this population have an unwanted effect of increasing, rather than decreasing, the risk of re-offending and that this might account for some of the differences in rates of re-offending by court outcomes that we observed.

We did not have access to general practitioner records of treatment for the cohort and this may have resulted in an incorrect assumption that some of those individuals identifying as receiving no treatment and management may, in fact, have received treatment and management. We had no detailed information on the type and quality of treatment and management received for those individuals who had a record of contact with a mental health service. However, we were able to disaggregate administrative contacts from actual clinical contact and this is one of the strengths of the current study. Future research could focus on the impact of specific treatment modalities to enable better elucidation of what works for this population in terms of reducing offending. Similarly, linking data such as ours to Medicare records held by the Commonwealth government would allow the impact of general practitioner contact and medication to be considered. As is the case in many longitudinal data-linkage studies, we did not have access to complete offence histories for the cohort and it is likely that some individuals had offence records that pre-dated the court outcome data that we had access to.

### Implications

Notwithstanding these considerations, overall our findings suggest that changes to current practices by the SCCLS and community mental health services might improve outcomes for those with psychosis appearing before the courts. Assuming that treatment and management is effective in reducing subsequent offending in those with psychosis, it may be worthwhile resourcing the community forensic mental health services to ensure that treatment and management is received by those with Section 32 treatment orders (i.e. self-initiated treatment). Interventions and incentives may need to be developed and explored to increase adherence.

Magistrates' behaviour may reflect their long-term observations and knowledge of different offender groups' behaviour in complying with treatment orders, such that they perceive those with drugs-related diagnoses as being less likely to respond to treatment. We were unable to conduct a separate analysis of treatment and management success or failure among those who had committed drug-related offences as only 5% of these individuals received a treatment order.

In conclusion, this study contributes to the existing literature on court diversion schemes for offenders who are mentally ill by demonstrating that offenders with a diagnosis of psychosis prior to their court appearance for the first principal offence were less likely to commit a second offence if they received a treatment order rather than a punitive sanction, such as a fine or good behaviour bond. It appears that those presented before the courts with either a drug-related offence or diagnoses of substance-related psychosis were less likely to receive a treatment order.
